# Biomarkers of Tobacco Exposure Decrease After Smokers Switch to an E-Cigarette or Nicotine Gum

**DOI:** 10.1093/ntr/nty140

**Published:** 2018-09-10

**Authors:** Elaine K Round, Peter Chen, Anthony K Taylor, Eckhardt Schmidt

**Affiliations:** 1 Scientific & Regulatory Affairs, RAI Services Company, Winston-Salem, NC; 2 AbbVie, North Chicago, IL

## Abstract

**Introduction:**

The aerosol composition of electronic cigarettes (ECs) suggests that exposure to toxicants during use is greatly reduced compared to exposure from combustible cigarettes (CCs).

**Methods:**

This randomized, parallel-group, clinical study enrolled smokers to switch to Vuse Solo (VS) Digital Vapor Cigarettes (Original or Menthol) or Nicorette 4 mg nicotine gum (NG) in a controlled setting. Subjects who smoked CCs *ad libitum* for 2 days during a baseline period were then randomized to *ad libitum* use of either VS or NG for 5 days. Biomarkers of 23 toxicants were measured in 24-hour urine samples and blood collected at baseline and following product switch.

**Results:**

A total of 153 subjects completed the study. Total nicotine equivalents decreased in all groups, but higher levels were observed in the VS groups compared to the NG groups, with decreases of 38% and 60%–67%, respectively. All other biomarkers were significantly decreased in subjects switched to VS, and the magnitude of biomarker decreases was similar to subjects switched to NG. Decreases ranged from 30% to greater than 85% for constituents such as benzene and acrylonitrile.

**Conclusions:**

These results indicate that exposure to toxicants when using VS is significantly reduced compared to CC smoking, and these reductions are similar to those observed with use of NG. Although statistically significantly decreased, nicotine exposure is maintained closer to CC smoking with VS use compared to NG use. This research suggests that use of VS exposes consumers to fewer and lower levels of smoke toxicants than CCs while still providing nicotine to the consumer.

**Implications:**

This is the first study to report changes in nicotine delivery and biomarkers of tobacco exposure following a short-term product switch from CCs to either an EC or NG in a controlled environment. The study shows that nicotine exposure decreased in both groups but was maintained closer to CC smoking with the EC groups. Biomarkers of tobacco combustion decreased to similar levels in both EC and gum groups.

## Introduction

Cigarette smoking is a leading cause of preventable death in the United States and has been associated with several types of diseases including cancer and heart disease.^1,2^ A continuum of risk of nicotine-containing products, as proposed by Kozlowski et al.,^3^ is the concept that nicotine-delivering products vary widely in the risks to the individual consumer and population health. This continuum has been explicitly adopted by the Strategic Dialogue on Tobacco Harm Reduction and embodied by the 2014 Surgeon General’s report.^2,4^ This framework clarifies that combustible cigarettes (CCs), on one end of the continuum, are the major cause of tobacco-related disease and are associated with the highest health risk. On the other end of the continuum are medicinal nicotine and noncombustible tobacco products including electronic cigarettes (ECs) that may contribute to reducing tobacco-related disease. Over the last several years, ECs have been increasingly used by smokers as an alternative to CCs.^5–9^ The Royal College of Physicians has stated that EC products are 95% less risky than CCs; however, the long-term effects of use will remain under study for years to come.^10^

Several studies have assessed the aerosol emitted from various brands of first-generation cig-alike EC products. Although constituents such as carbonyls and metals are detected in these products, the levels are generally many times lower than the levels observed in CC smoke.^11–15^ This research suggests that exposure to the harmful and potentially harmful constituents (HPHCs) found in cigarette smoke would be greatly reduced when CC smokers switch to an EC product.

ECs were brought under regulatory authority of the US Food and Drug Administration (FDA) on August 8, 2016, and will require regulatory filing and marketing authorization to be legally sold in the United States after the initial compliance period ends. In its *Premarket Tobacco Product Applications for Electronic Nicotine Delivery Systems* Draft Guidance, the FDA Center for Tobacco Products states that marketing authorization will be granted if an electronic nicotine delivery system product, which includes ECs, is demonstrated to be appropriate for the protection of public health. Among the many types of research suggested to support such an application, an assessment of biomarkers of tobacco exposure and harm in consumers of these products is recommended.^16^ In addition, experts in the field have proposed research agendas to better understand the health effects of EC products.^17,18^ Biomarkers of tobacco exposure have been extensively researched and many biomarkers exist that distinguish among smokers, smokeless-tobacco consumers, and nontobacco consumers.^19,20^ Several researchers have made recommendations to apply these biomarkers to tobacco product regulation and disease prevention.^21–23^

There has been limited exploration to date of biomarkers of tobacco exposure associated with the use of ECs. Study types have included cross-sectional studies with varying cohort types including smokers, exclusive EC users of differing product generations and durations of use, dual users, and nicotine replacement therapy users; a short-term in-clinic switching study; and two longitudinal studies of smokers who switched to ECs in their natural environments. In general, these studies have shown that biomarker levels are markedly lower when consumers use ECs compared to CCs.^24–31^

This study evaluated the changes in nicotine biomarkers, product use, and biomarkers of 22 carcinogens and toxicants found in cigarette smoke after smokers switch to *ad libitum* use of Vuse Solo (VS) Digital Vapor Cigarettes (Original or Menthol) for 5 days. A group who switched to *ad libitum* use of nicotine gum (NG) was included for qualitative comparison to the maximum possible reductions in biomarkers of tobacco exposure over the study period while still allowing for nicotine use. This is the first study to report changes in nicotine delivery and biomarkers of tobacco exposure following a short-term product switch from CCs to either ECs or NG in a controlled environment.

## Methods

This was a randomized, controlled, open-label, parallel group study conducted by DaVita Clinical Research at one site in Minneapolis, Minnesota, between January and May 2015. The study was approved by Chesapeake Institutional Review Board (Columbia, Maryland) in November 2014 and was performed in accordance with the principles of the Declaration of Helsinki 2013. Registration on *www.clinicaltrials.gov* (Identifier number NCT02323438) occurred on December 18, 2014. Written informed consent was obtained from each subject before study procedures were performed.

### Participants

Generally healthy males and females, 21–60 years of age, inclusive, who reported smoking at least 10 combustible, filtered, menthol or non-menthol cigarettes per day and reported smoking their first cigarette within 30 minutes of waking were included in the study. In addition, potential participants had to be willing to switch from their usual brand (UB) cigarettes to VS Original flavor, VS Menthol flavor, or NG while in clinic. Subjects with controlled, chronic health conditions were included at the discretion of the investigator; however, diabetic subjects were excluded. A total of 385 subjects were screened for the study, and 153 subjects completed all study activities.

### Test Products and Product Use

VS Digital Vapor Cigarettes were introduced commercially by R.J. Reynolds Vapor Company in March 2013. The product is a first-generation cig-alike product composed of a battery, heating element, microchip, sensor, and a cartridge containing e-liquid composed of propylene glycol, glycerin, nicotine, flavorings, and water. During use, the heating element aerosolizes the liquid in the cartridge and produces a puff of aerosol that contains aerosol-forming excipients (propylene glycol and glycerin) and nicotine. A microchip in the cartridge tracks puffing activation time to prevent depletion of e-liquid. Power wattage is the most informative parameter of an EC with respect to the heating of the e-liquid. The effective power to the VS cartridge is controlled to approximately 3 W during a puff. The two brand styles used in this study include VS Original, a tobacco flavor, and VS Menthol. Both brand styles contain approximately 600 µL of a 4.8% nicotine e-liquid, or approximately 29 mg of nicotine.

Nicorette nicotine polacrilex gum (GlaxoSmithKline Consumer Healthcare, LP, Philadelphia, PA) is commercially available in 2 and 4 mg strengths. The 4-mg NG was chosen for use in this study in order to include smokers who typically have higher levels of nicotine exposure. Instructions on the package state: “If you smoke your first cigarette within 30 minutes of waking up, use 4 mg nicotine gum.” The White Ice Mint flavor was provided for use in this study. Subjects received written instructions for use based on the Nicorette gum package label. Nicorette gum was the current market leader among oral nicotine replacement therapies at the time this study was conducted.

All subjects provided their own UB cigarettes for use during the baseline period. UB cigarettes were collected by site staff at check-in on day −3 and dispensed to subjects one at a time upon request until 11:00 pm on day −3 and from 07:00 am to 07:30 pm on day −2. Following randomization, subjects used VS or NG *ad libitum* from 07:00 am to 11:00 pm on days 1, 2, 3, and 4. On day 5, *ad libitum* use occurred from 07:00 am to 07:30 pm. Product use ended earlier on Days −2 and 5 to start a 12-hour nicotine abstinence period in preparation for nicotine pharmacokinetic assessments on Days −1 and 6 (data to be reported in a separate publication). Days −1 and 6 involved a short duration of product use starting no earlier than 07:30 am, followed by nicotine abstinence and blood collection over a 6-hour period. After completion of the 6 hours, subjects on Day −1 smoked *ad libitum* until 11:00 pm, and subjects on Day 6 were discharged from the study following completion of final safety procedures.

### Study Design

A full schematic of the study design can be found in Supplementary Figure 1. Potential subjects completed a prescreening telephone interview and one screening visit to assess eligibility within 30 days of study enrollment on Day −3. On Day −3, eligible subjects were enrolled in the study and started a 9-day in-clinic residence. Baseline assessments during smoking of subjects’ UB cigarettes occurred for the first 3 days (Day −3 through Day −1). On Day 1, smokers were randomized to one of four cohorts.

Smokers of non-menthol cigarettes were randomized to one of two cohorts:

• Cohort 1: EC − VS Original, or
• Cohort 2: NG

Smokers of menthol cigarettes were randomized to one of two cohorts:

• Cohort 3: EC − VS Menthol, or
• Cohort 4: NG

Post–product switch assessments occurred for 6 days (Day 1 through Day 6). Upon completion of study procedures on Day 6, subjects were discharged from the clinic. The Fagerström Test for Nicotine Dependence and a demographic questionnaire were administered to all potential subjects at the screening visit.^32^

### Biological Sample Collection

Whole blood samples were collected at approximately 07:00 pm on Days −2, 1, 3, and 5 for measurement of carboxyhemoglobin percent saturation. Measurements were performed at LabCorp (Burlington, NC and Minneapolis, MN) using a carbon monoxide oximeter to spectrophotometrically measure carboxyhemoglobin and hemoglobin.

Plasma was collected on Days −2, 1, 3, and 5 at approximately 07:00 am (before product use began each day), 01:00 pm, and 07:00 pm for measurement of nicotine and cotinine. Additional plasma samples were collected on Days −1 and 6 for nicotine pharmacokinetic analysis just before and for 6 hours following the start of a single *ad libitum* use period (data to be reported elsewhere). Plasma samples were processed and aliquoted within 90 minutes of collection and stored at −70°C until shipment for analysis.

Urine samples were collected for 24-hour periods starting at 07:30 pm on Days −3 and 4 and ending at 07:30 pm on Days −2 and 5. Urine was stored at 4°C until collection was complete. Total 24-hour volumes were recorded, and samples were aliquoted and stored at −70°C until shipment for analysis.

### Urine Mutagenicity

Aliquots of 24-hour urine samples were shipped on dry ice to Covance Laboratories Limited (Harrogate, UK) for assessment of urine mutagenicity using a modified Ames assay. *Salmonella typhimurium* strain YG1024 with S9 for metabolic activation was used for assessment, and the assay and analysis were performed as described in Krautter et al., 2014.^33^

### Biomarker Analysis

Urinary biomarker analysis was performed by ABF GmbH (Munich, Germany), and plasma nicotine and cotinine analysis was performed by Celerion, Inc. (Lincoln, NE). Methods are generally as described in Theophilus et al.^34^ and Round et al.,^35^ with one exception. The calculation of total nicotine equivalents includes unconjugated nicotine, unconjugated cotinine, unconjugated trans-3′-hydroxycotinine, nicotine-N-glucuronide, cotinine-N-glucuronide, *trans*-3′-hydroxycotinine-O-glucuronide, cotinine-N-oxide, nicotine-N-oxide, norcotinine, nornicotine, and 4-hydroxy-4-(3-pyridyl)-butanoic acid.^36^

Results for each biomarker were reported by the lab as a concentration. Total daily excretion yields for each biomarker were determined by multiplying the observed biomarker concentration by the total urine volume for each 24-hour collection to obtain biomarker mass/24 h. When observed concentrations were below the limit of quantification, a value of ½ limit of quantification was imputed and used for determination of 24-hour totals. When multiple metabolites were included in the calculation of total constituent equivalents (eg, nicotine, acrylamide, and naphthalene), the individual metabolites (mass/24 h) were converted to molar equivalents of the parent compound and summed. The full list of biomarkers can be found in Tables 2 and 3.

### Statistical Analysis

A sample size of 35 completed subjects per cohort was estimated to provide 80% power to detect a 25% reduction with a Bonferroni-adjusted *p* value of .05/36. The powering for the full clinical study used a Bonferroni-adjusted significance level that included 36 comparisons, not all of which are presented here. Data from previous studies were used for the sample-size determination. Up to 41 subjects per cohort were enrolled to ensure 35 completed the study. Demographic, Fagerström Test for Nicotine Dependence, and product-use descriptive statistics were calculated for all randomized subjects. Biomarker data were summarized for all subjects who completed the study. Percent changes were calculated as the percent difference between the mean biomarker values from baseline to post–product switch.

A two-sided paired *t* test was used to determine the significance of differences between Day −2 and Day 5 urinary total nicotine equivalents results within cohorts. A two-sided test was employed here because neither an increase nor a decrease in nicotine exposure could be predicted before conducting the study. A one-sided paired *t* test was used to determine the significance of differences between Day −2 and Day 5 biomarker results within cohorts for all other urinary biomarkers and blood carboxyhemoglobin. A one-sided test was employed here because these biomarkers were all expected to decrease based on chemical analysis of the aerosol produced by VS during machine puffing. All calculations were performed using SAS Version 9.1 or higher. All *p* values were adjusted using a Bonferroni step-down method including 36 comparisons to maintain an overall significance level of 0.05.

## Results

A total of 385 subjects were screened for the study, of which 162 were enrolled on Day −3, and 158 were randomized on Day 1. Of those randomized, 38, 39, 40, and 41 subjects were randomized to Cohorts 1, 2, 3, and 4, respectively. Five subjects withdrew consent after randomization, resulting in 153 subjects who completed the study, with 37, 38, 38, and 40 subjects completing the study in Cohorts 1, 2, 3, and 4, respectively.

Table 1 summarizes the demographics and baseline characteristics of subjects by cohort. Subjects generally had similar Fagerström Test for Nicotine Dependence scores, with means of 6.0–6.3 per cohort, and smoked similar numbers of cigarettes per day at baseline. Consistent with the US smoking population, non-menthol smokers in this study were predominantly non-Hispanic whites. A higher percentage of African American smokers smoke menthol cigarettes, and the menthol smokers in this study were predominantly black/African American, consistent with these demographics.^38^

**Table 1.** Demographics and Baseline Characteristics (*n* [%]), or mean ± SD)

	NM smoker—VS original (*N* = 38)	NM smoker—nicotine gum (*N* = 39)	M smoker—VS menthol (*N* = 40)	M smoker—nicotine gum (*N* = 41)
Age, mean ± SD	41.63 ± 11.22	40.18 ± 11.44	42.55 ± 10.87	41.46 ± 10.00
Gender, *n* (%)				
Female	11 (28.9)	14 (35.9)	15 (37.5)	11 (26.8)
Male	27 (71.1)	25 (64.1)	25 (62.5)	30 (73.2)
Ethnicity, *n* (%)				
Hispanic or Latino	1 (2.6)	0	0	1 (2.4)
Non-Hispanic or Latino	37 (97.4)	39 (100)	40 (100)	40 (97.6)
Race, *n* (%)				
American Indian or Alaskan Native	0	0	4 (10.0)	1 (2.4)
Asian	0	0	0	0
Black or African American	14 (36.8)	13 (33.3)	25 (62.5)	29 (70.7)
Native Hawaiian or Other Pacific Islander	0	0	0	0
White	21 (55.3)	25 (64.1)	11 (27.5)	6 (14.6)
Multiple	2 (5.3)	0	0	5 (12.2)
Other	1 (2.6)	1 (2.6)	0	0
Highest level of school completed				
Grade school	0	0	0	0
High school (grades 9–11)	3 (7.9)	1 (2.6)	4 (10.0)	5 (12.2)
High school graduate or GED	16 (42.1)	15 (38.5)	11 (27.5)	13 (31.7)
Technical school	5 (13.2)	3 (7.7)	6 (15.0)	3 (7.3)
Some college	8 (21.1)	15 (38.5)	14 (35.0)	13 (31.7)
College graduate	6 (15.8)	5 (12.8)	4 (10.0)	6 (14.6)
Graduate school	0	0	0	0
Decline to answer	0	0	1 (2.5)	1 (2.4)
FTND, mean ± SD	6.0 (1.5)	6.3 (1.4)	6.0 (1.5)	6.2 (1.4)

FTND = Fagerström Test for Nicotine Dependence; GED = General Education Diploma; M = Menthol; NM = Non-menthol; SD = standard deviation; VS = Vuse Solo.

### Nicotine Biomarkers

Mean total nicotine equivalents in 24-hour urine samples decreased 38% in both VS groups and decreased 60% and 67% in the gum groups of non-menthol and menthol smokers, respectively (Table 2). Reductions in plasma nicotine and cotinine concentrations measured at 07:00 pm on Days −2 and 5 mirrored the reductions in total urinary nicotine equivalents in all groups.

**Table 2.** Biomarkers of Nicotine Exposure, Mean ± SD at Baseline and Day 5 and Percent Change

Nicotine biomarker	Baseline (mean ± SD)	Day 1 (mean ± SD)	Day 3 (mean ± SD)	Day 5 (mean ± SD)	Baseline to Day 5 percent change
Urinary nicotine equivalents (mg/24 h)					
NM smoker—VS original^a^	20.9 ± 7.6	—	—	12.9 ± 9.8	−38.3
NM smoker—gum^b^	19.5 ± 5.7	—	—	7.9 ± 6.1	−59.7
M smoker—VS menthol^c^	21.5 ± 6.9	—	—	13.4 ± 8.8	−37.8
M smoker—gum^d^	21.7 ± 7.8	—	—	7.2 ± 4.3	−66.7
Plasma cotinine at 07:00 pm (ng/mL)					
NM smoker—VS original^a^	269 ± 108	159 ± 83	160 ± 122	183 ± 153	−32.0
NM smoker—gum^b^	264 ± 94	162 ± 75	113 ± 78	117 ± 95	−55.7
M smoker—VS menthol^c^	311 ± 114	201 ± 117	186 ± 123	211 ± 148	−32.2
M smoker—gum^d^	317 ± 111	184 ± 71	122 ± 74	110 ± 77	−65.3
Plasma nicotine at 07:00 pm (ng/mL)					
NM smoker—VS original^a^	19.2 ± 9.0	6.5 ± 5.9	9.6 ± 8.9	11.5 ± 10.4	−40.1
NM smoker—gum^b^	19.0 ± 8.5	6.4 ± 4.3	5.2 ± 5.2	6.0 ± 5.4	−68.4
M smoker—VS menthol^c^	20.3 ± 8.4	7.6 ± 4.2	10.5 ± 8.2	13.0 ± 9.8	−36.0
M smoker—gum^d^	21.9 ± 8.3	5.6 ± 4.3	4.4 ± 3.8	5.3 ± 4.2	−75.8

Statistical significance for change in urinary nicotine equivalents was determined using a two-sided paired *t* test and adjusted using a Bonferroni step-down method. All changes from baseline to Day 5 were statistically significant (*p* < .05). Statistical significance for trend in plasma cotinine and nicotine was determined using mixed models with repeated measures for analysis of day effect. *p* values were adjusted using a step-down Bonferroni method. All trends were statistically significant (*p* < .05). CPD = cigarettes per day; M = Menthol; NM = Non-menthol; SD = standard deviation; VS = Vuse Solo.

^a^ *n* = 37.

^b^ *n* = 38.

^c^ *n* = 38.

^d^ *n* = 40.

### Biomarkers of Exposure

Biomarkers of toxicants decreased 30%–99% for all groups, and generally decreased by similar amounts whether subjects were switched to an e-cigarette or NG (Table 3).

**Table 3.** Biomarkers of Tobacco Smoke Exposure in 24-hour Urine and Whole Blood (Mean ± SD) and Percent Change of the Mean Biomarker Amounts From Baseline to Day 5

		Non-menthol smoker—VS original (*n* = 37)			Non-menthol smoker—gum (*n* = 38)			Menthol smoker—VS menthol (*n* = 38)			Menthol smoker—gum (*n* = 40)		
Biomarker	Toxicant	Baseline (M ± SD)	Day 5 (M ± SD)	% change	Baseline (M ± SD)	Day 5 (M ± SD)	% change	Baseline (M ± SD)	Day 5 (M ± SD)	% change	Baseline (M ± SD)	Day 5 (M ± SD)	% change
COHb (% saturation)^a^	Carbon monoxide	5.8 ± 1.6	1.4 ± 0.6	−75.3	5.4 ± 1.4	1.3 ± 0.4	−75.0	6.0 ± 1.7	1.4 ± 0.4	−77.1	5.7 ± 1.8	1.4 ± 0.5	−76.1
SPMA (µg/24 h)^b^	Benzene	3.7 ± 2.2	0.4 + 0.2	−89.7	4.4 ± 3.8	0.4 ± 0.3	−90.1	3.9 ± 2.0	0.4 ± 0.2	−89.0	4.4 ± 4.8	0.5 ± 0.2	−89.3
3-HPMA (µg/24 h)^b^	Acrolein	2052.8 ± 1274.3	605.6 ± 291.2	−70.5	1828.9 ± 518.5	512.5 ± 192.0	−72.0	2065.1 ± 790.9	598.3 ± 238.3	−71.0	1983.7 ± 632.6	623.5 ± 274.9	−68.6
HMPMA (µg/24 h)^b^	Crotonaldehyde	578.2 ± 327.2	129.9 ± 75.7	−77.5	537.8 ± 186.2	118.9 ± 37.6	−77.9	564.1 ± 202.0	128.4 ± 58.2	−77.2	549.1 ± 204.8	128.9 ± 56.5	−76.5
MHBMA (µg/24 h)^b^	1,3-butadiene	4.9 ± 3.2	2.2 ± 2.6	−55.5	5.2 ± 2.9	1.9 ± 2.0	−63.4	4.2 ± 2.5	1.9 ± 1.8	−56.0	4.2 ± 2.2	2.6 ± 2.5	−37.7
CEMA (µg/24 h)^b^	Acrylonitrile	261.2 ± 187.1	36.8 ± 21.7	−85.9	226.9 ± 72.3	29.0 ± 13.6	−87.2	254.0 ± 94.6	36.5 ± 19.8	−85.6	246.1 ± 106.2	34.7 ± 16.6	−85.9
HEMA (µg/24 h)^b^	Ethylene oxide	16.4 ± 9.2	6.2 ± 3.1	−62.3	20.0 ± 17.5	7.9 ± 6.1	−60.4	16.7 ± 11.8	7.7 ± 4.6	−53.9	17.5 ± 11.2	9.4 ± 4.7	−46.0
NNAL-T (ng/24 h)^b^	NNK	603.1 ± 428.9	249.4 ± 165.3	−58.7	483.8 ± 313.6	176.7 ± 113.1	−63.5	532.3 ± 365.6	239.7 ± 155.4	−55.0	503.1 ± 317.4	201.4 ± 115.8	−60.0
NNN-T (ng/24 h)^b^	NNN	21.4 ± 17.1	2.7 ± 2.4	−87.4	27.8 ± 18.8	3.2 ± 4.9	−88.6	32.5 ± 32.9	2.7 ± 1.2	91.8	24.5 ± 15.7	2.5 ± 1.2	−89.8
NAT-T (ng/24 h)^b^	NAT	303.8 ± 290.1	3.9 ± 7.9	−98.7	295.8 ± 223.4	2.4 ± 1.0	−99.2	264.5 ± 191.3	5.6 ± 7.9	−97.9	286.1 ± 225.3	4.6 ± 9.0	−98.4
NAB-T (ng/24 h)^b^	NAB	54.5 ± 47.1	5.8 ± 2.7	−89.5	51.7 ± 34.3	6.1 ± 2.6	−88.3	47.5 ± 30.7	6.4 ± 2.2	−86.5	46.2 ± 33.4	6.9 ± 5.1	−85.0
1-AN (ng/24 h)^b^	1-aminonaphthalene	109.3 ± 43.0	4.5 ± 2.6	−95.5	103.1 ± 34.8	4.3 ± 1.7	−95.8	106.2 ± 41.5	5.3 ± 2.6	−95.0	109.1 ± 42.3	7.4 ± 13.5	−94.2
2-AN (ng/24 h)^b^	2-aminonaphthalene	27.4 ± 13.9	2.6 ± 1.4	−90.4	27.7 ± 10.4	2.5 ± 1.4	−90.9	29.2 ± 12.6	2.4 ± 0.7	−91.9	29.5 ± 12.7	2.5 ± 1.4	−91.5
3-ABP (ng/24 h)^b^	3-aminobiphenyl	10.6 ± 5.0	2.8 ± 1.4	−74.0	9.6 ± 3.9	2.1 ± 1.3	−78.0	10.1 ± 5.0	2.2 ± 1.0	−78.6	10.4 ± 5.3	2.0 ± 0.9	−80.6
4-ABP (ng/24 h)^b^	4-aminobiphenyl	21.2 ± 8.8	7.8 ± 3.4	−63.5	22.6 ± 9.3	7.2 ± 3.8	−68.3	22.4 ± 8.1	6.1 ± 2.6	−73.0	23.0 ± 8.0	6.6 ± 2.3	−71.4
o-toluidine (ng/24 h)^b^	o-toluidine	259.3 ± 292.8	109.9 ± 108.4	−57.6	203.4 ± 75.0	97.8 ± 75.1	−51.9	203.6 ± 66.1	90.2 ± 33.3	−55.7	203.0 ± 68.3	96.0 ± 44.7	−52.7
Naphthalene equivalents (µg/24 h)^b^	Naphthalene	34.6 ± 14.1	5.7 ± 3.5	−83.6	33.8 ± 12.7	5.4 ± 2.5	−83.9	36.2 ± 13.0	10.6 ± 14.4	−70.1	42.3 ± 29.0	11.8 ± 18.3	−72.0
3-OH-B[a]P (pg/24 h)^b^	Benzo[a]pyrene	258.1 ± 325.2	93.6 ± 73.6	−63.8	621.3 ± 2443.2	133.4 ± 171.6	−78.5*	270.2 ± 359.2	81.1 ± 52.9	−70.0	189.6 ± 157.6	104.5 ± 110.0	−44.9
2-OH-fluorene (µg/24 h)^b^	Fluorene	1.7 ± 1.0	1.2 ± 0.9	−30.4	1.7 ± 0.8	1.0 ± 0.4	−43.0	1.7 ± 0.8	1.1 ± 0.6	−35.7	1.7 ± 0.8	1.1 ± 0.6	−34.2
1-OH-pyrene (ng/24 h)^b^	Pyrene	503.5 ± 260.4	183.9 ± 128.5	−63.5	679.4 ± 1026.1	336.1 ± 731.0	−50.5	568.8 ± 407.9	186.5 ± 180.6	−67.2	529.3 ± 390.7	199.9 ± 158.9	−62.2
Acrylamide equivalents (µg/24 h)^b^	Acrylamide	106.0 ± 30.9	53.1 ± 19.4	−50.0	98.3 ± 30.1	49.7 ± 15.9	−49.2	108.8 ± 36.3	49.7 ± 16.0	−54.3	113.3 ± 34.6	55.9 ± 16.8	−50.6
Thiocyanate (µmol/24 h)^b^	Hydrogen cyanide	171.3 ± 103.3	103.8 ± 45.7	−39.4	143.8 ± 68.3	101.6 ± 46.8	−29.3	186.8 ± 114.2	119.5 ± 54.5	−36.0	152.9 ± 81.9	108.5 ± 48.0	−29.0
Urine mutagenicity (Revertants/10^3^/24 h)^b^	General measure of mutagenic properties of urine	272.5 ± 183.5	32.4 ± 25.8	−88.1	226.0 ± 155.5	40.2 ± 46.0	−82.2	296.6 ± 215.7	29.9 ± 24.9	−90.0	344.7 ± 290.4	38.6 ± 27.5	−88.8

Statistical significance was determined within cohort using a one-sided paired *t* test and was adjusted using a Bonferroni step-down method. All changes from baseline to Day 5 were statistically significant (*p* < .05) except as indicated by *. 1-AN = 1-aminonaphthalene; 2-AN = 2-aminonaphthalene; 3-ABP = 3-aminobiphenyl; 3-HPMA = 3-hydroxypropyl mercapturic acid; 3-OH-B[a]P = 3-OH-benzo[a]pyrene; 4-ABP = 4-aminobiphenyl; CEMA = 2-cyanoethylmercapturic acid; COHb = carboxyhemoglobin; HEMA = 2-hydroxyethylmercapturic acid; HMPMA = 3-hydroxy-1-methylpropylmercapturic acid; M = mean; MHBMA = monohydroxybutyl mercapturic acid; NAB = *N*’-nitrosoanabasine; NAB-T = free plus *N*-glucuronidated (total) *N*’-nitrosoanabasine; NAT = *N*’-nitrosoanatabine; NAT-T = free plus *N*-glucuronidated (total) *N*’-nitrosoanatabine; NNAL-T = free plus *N*-glucuronidated (total) 4-(methylnitrosamino)-1-(3-pyridyl)-1-butanol; NNK = 4-(methylnitrosamino)-1-(3-pyridyl)-1-butanone; NNN = *N*’-nitrosonornicotine; NNN-T = free plus *N*-glucuronidated (total) *N*’-nitrosonornicotine; SD = standard deviation; SPMA = *S*-phenylmercapturic acid; VS = Vuse Solo.

^a^Measured in whole blood.

^b^Measured in urine.

Carboxyhemoglobin, both a biomarker of disease risk and a measure of CC cessation, decreased approximately 75% in all groups. Changes in other vapor-phase biomarkers decreased 38%–90% in all groups. In addition, tobacco-specific nitrosamines decreased 55%–99%, aromatic amines decreased 52%–96%, and polycyclic aromatic hydrocarbons decreased 30%–84% in all groups. Biomarkers of two constituents with longer half-lives, acrylamide and hydrogen cyanide, decreased approximately 50% and 30%–40%, respectively, consistent with expected decreases based on results of similar studies with a smoking-cessation cohort.^30,33,38,39^

All biomarker decreases were statistically significant (*p* < .05) in all cohorts, with the exception of the decrease observed in 3-hydroxy-benzo[a]pyrene in the non-menthol smoker gum group. One subject in that cohort showed a baseline value of nearly six standard deviations above the mean. In addition, another subject showed an approximately 20-fold higher value at Day 5 compared to Day −2. These results created a large variation among subjects, which decreased the sensitivity of the *t* test. Therefore, despite a 78.5% decrease in 3-hydroxy-benzo[a]pyrene, the difference was not statistically significantly different in the non-menthol smoker gum group.

### Product Use

Product use amounts for each full day of *ad libitum* use are summarized in Table 4, including the number of cigarettes smoked over a 24-hour period at baseline and the amounts of e-liquid and gum used per day for 5 days after product switch. Cigarettes per day at baseline were similar across the four cohorts, ranging from means of 14.0 to 14.5. Following randomization, subjects chose to use the products to which they were assigned, as evidenced by the product-use results for individual subjects. Among subjects randomized to VS who completed the study, 75 of 77 subjects used at least 0.10 g of e-liquid per day on at least 3 of 5 days. Among subjects randomized to NG who completed the study, all used at least one piece on at least 3 of 5 days.

**Table 4.** Daily Consumption of Cigarettes, E-Liquid (g), and Nicotine Gum (Pieces)

	NM smoker—VS original (mean gram e-liquid ± SD) *N* = 38^a^	NM smoker—nicotine gum (mean pieces ± SD) *N* = 39^b^	M smoker—VS menthol (mean gram e-liquid ± SD) *N* = 40^c^	M smoker—nicotine gum (mean pieces ± SD) *N* = 41^d^
Baseline CPD (mean ± SD)	14.0 ± 4.0	14.4 ± 3.7	14.5 ± 4.6	14.3 ± 2.7
Post–product switch				
Day 1	0.26 ± 0.23	5.6 ± 2.2	0.28 ± 0.21	4.8 ± 1.9
Day 2	0.36 ± 0.30	4.7 ± 2.3	0.38 ± 0.27	4.5 ± 2.3
Day 3	0.42 ± 0.30	5.0 ± 3.0	0.42 ± 0.30	4.6 ± 2.7
Day 4	0.43 ± 0.32	4.6 ± 3.0	0.44 ± 0.32	4.4 ± 2.6
Day 5	0.40 ± 0.30	4.4 ± 2.5	0.42 ± 0.29	3.9 ± 2.4

Daily nicotine intake for VS users may be calculated by multiplying the daily 4.8% nicotine e-liquid mass values above by 0.048. CPD = cigarettes per day; M = Menthol; NM = non-menthol; SD = standard deviation; VS = Vuse Solo.

^a^Includes a partial day of use for one subject on Day 5 due to withdrawal of consent.

^b^Final *n* = 38 due to withdrawal of consent for one subject on Day 4.

^c^Final *n* = 38 due to withdrawal of consent for one subject on Day 3 and one subject on Day 4.

^d^Includes a partial day of use for one subject on Day 5 due to withdrawal of consent.

The mean daily amounts of e-liquid used by the VS groups increased from Day 1 to Day 3 and then the amounts used on Days 3, 4, and 5 were relatively consistent. *Ad libitum* use was permitted for a shorter period of time on Day 5 than on the other days post–product switch due to the start of the 12-hour abstinence at 07:30 pm, and the lower average amount of use on that day likely reflects this. In contrast to e-liquid use, average daily use of gum was relatively constant throughout the study: non-menthol smokers used approximately 4.5–6 pieces per day, and menthol smokers used approximately 4–5 pieces per day.

### Adverse Events

Eighty-three adverse events were reported among randomized subjects during this study; 35 in the VS groups and 48 in the gum groups, all of which were mild or moderate. Of these, 43 were determined by the investigator to be possibly or definitely related to product use: 17 in the VS groups and 26 in the gum groups. The most common adverse events reported by the VS groups were headache, nausea, and cough. The most common adverse events reported by the gum groups were dyspepsia, hiccups, and oropharyngeal pain.

## Discussion

This study was designed to evaluate changes in nicotine uptake and exposure to toxicants of tobacco combustion, and to understand product-use behavior after a short-term switch from CCs to either VS ECs (non-menthol or menthol) or NG. Both products were generally well tolerated by subjects.

Nicotine uptake was higher during baseline than in either of the VS groups or NG groups. Although statistically significant decreases in total nicotine equivalents occurred in all groups, subjects in the VS groups reduced their nicotine uptake by a lesser extent than subjects in the gum groups (a qualitative comparison of approximately 40% versus approximately 60%, respectively). Plasma nicotine and cotinine concentrations observed at 07:00 pm on Day −2 and Day 5 showed similar results (Table 2). Although substantial nicotine uptake occurred in both the VS and gum groups, these results indicate that neither product, when used *ad libitum*, resulted in the same level of nicotine uptake as CCs under the conditions of this study.

In general, large, significant reductions were seen in biomarkers of carcinogens and toxicants. These results indicate that exposure to many HPHCs present in cigarette smoke is greatly reduced by using VS and that those reductions are similar to the reductions that occur when a smoker switches to NG. Several biomarkers measured in this study are known to have longer elimination half-lives, including 4-(methylnitrosamino)-1-(3-pyridyl)-1-butanol (NNAL), acrylamide equivalents, and thiocyanate, and the smaller reductions observed for those are as expected.

Biomarker reductions in all groups were similar to the reductions observed in tobacco-abstinent groups of studies of a similar design, with two exceptions. Biomarkers of fluorene and 1,3-butadiene decreased less than expected in all groups, with decreases of approximately 35% and 55%, respectively, compared to 70% and 90%, respectively, in smokers randomized to tobacco abstinent groups in other studies.^30,33,38,39^ Although less than expected, the reductions here were consistent across all groups, which suggests that the results were not related to VS or NG use. An influence such as an unknown environmental exposure may have occurred equally for all groups, preventing reductions to levels observed in other studies.

The results for 3-OH-B[a]P generally showed larger variability across cohorts than other biomarkers. This is especially so for the non-menthol smoker gum cohort, which resulted in a nonstatistically significant decrease after product switch. This variability is largely due to 52% of subjects across all cohorts whose urinary B[a]P concentrations were measured as below the limit of quantification of 50 fg/mL for the method at baseline while smoking CCs. Similarly, 79% of subjects across all cohorts showed 3-OH-B[a]P levels below the limit of quantification after product switch; therefore, the change in mass/24 h for subjects with values below the limit of quantification for both time points is dependent on the volume of the urine collected. Increasing the sensitivity of the method may not be feasible because it is already validated to a very low level. Therefore, although B[a]P has been determined to be an HPHC by the FDA Center for Tobacco Products, and a metabolite unique to that compound exists, it may not be an appropriate biomarker of tobacco exposure due the very low levels detected in the urine of smokers.

The results presented here are similar to results observed in other studies that assessed biomarkers either after a 5-day switch, a 4- to 12-week switch, or in cross-sectional studies in which subjects reported short or longer-term EC use.^24–31^ Although the study designs differed, the biomarker differences between exclusive EC use and CC use were similar for those biomarkers with short elimination half-lives. Differences were observed between the studies that assessed short-term versus longer-term switching in results for NNAL, which were expected given the long elimination half-life of that biomarker, as discussed earlier.

Product-use patterns reported here showed similarity between the VS Original and Menthol groups. Both groups used an average of approximately 0.26–0.28 g of e-liquid on Day 1 (Table 4). Day 2 showed similar increases in use for both groups, with an average e-liquid use of approximately 0.36–0.38 g. Increase in e-liquid use was similar again on Day 3 at an average of approximately 0.42–0.44 g. Average use remained similar for both groups and similar to Day 3 use on Days 4 and 5. Although product use was slightly less for both groups on Day 5, participants were permitted to use product for 3.5 fewer hours than on Days 1–4. These results suggest that acclimation to the new product occurred over the first 2 days and that subjects reached their typical product use on Day 3 that continued through Day 5. Further research will be needed to confirm whether use after a 3-day acclimation period reflects how an EC consumer may use the product long term.

Average daily gum use was slightly less than the average reported use by smokers during their first week of cessation: approximately five pieces per day here compared to 7.7 pieces reported by Shiffman et al.,^40^ but was greater than the reported average of 3.2 pieces per day after 24 weeks of cessation.

This study was intended to determine the maximum biomarker reductions possible when smokers fully switched to VS. Several limitations of this study exist. First, the study was conducted in-clinic and did not examine subjects in their normal environments, which would allow for a more realistic picture of their choice of product use. Second, a continue-smoking cohort was not included, which would address whether subjects’ UB cigarettes per day might change in clinic. In addition, this study recruited smokers who were not postponing a decision to quit smoking; therefore, subjects may not have been highly motivated to switch to a product other than CCs.

This was a short-term study that focused on acute reductions in biomarkers with generally short elimination half-lives. The biomarkers measured were generally representative of smoke constituents to demonstrate how a switch from smoking to VS would affect exposure. In fact, 20 of the 23 biomarkers measured here represent compounds found on FDA’s established list of HPHCs found in tobacco products and tobacco smoke.^37^ In addition, 11 of the biomarkers measured are representative of constituents included on the list of 29 HPHCs that FDA recommends for analysis in electronic nicotine delivery system aerosols per their *Premarket Tobacco Product Applications for Electronic Nicotine Delivery System* Draft Guidance.^16^ Longer-term studies to assess biomarkers of potential harm and health effects will be important to understand the changes in health risk associated when smokers switch to VS, and ECs in general, for longer periods of time. Such studies might also include EC-specific biomarkers, such as nickel and chromium, as they become qualified to distinguish among tobacco-use groups.^41^

In summary, this study examined nicotine exposure, product-use patterns, and biomarkers of carcinogens and toxicants in smokers who exclusively switched to VS or NG for 5 days in a controlled setting. Results indicate that exposure to toxicants from VS use are significantly reduced and appear to be reduced to the same degree as seen with NG use, and adds to the research that suggests use of ECs, represented here by VS, exposes consumers to fewer and lower levels of smoke toxicants than CCs.

## Supplementary Material

Supplementary data are available at *Nicotine & Tobacco Research* online.

## Funding

This study was funded by R.J. Reynolds Vapor Company through R.J. Reynolds Tobacco Company.

## Declaration of Interests

ER, PC, and ES are full-time employees of RAI Services Company. AT was a full-time employee of RAI Services Company at the time this study was conducted. RAI Services Company is a wholly owned subsidiary of Reynolds American Inc., which is a wholly owned subsidiary of British American Tobacco plc.

## Supplementary Material

nty140_suppl_supplementary_Information
