# Supplementary material for: Biomarkers of Tobacco Exposure Decrease After Smokers Switch to an E-Cigarette or Nicotine Gum
Source: Nicotine Tob Res. 2018 Sep 10;21(9):1239–47. doi: 10.1093/ntr/nty140 (PMC6698949; doi:10.1093/ntr/nty140)
Supplement: nty140_suppl_supplementary_Information [file nty140_suppl_supplementary_information.docx]

Supplemental Information

Figure 1. Study Design


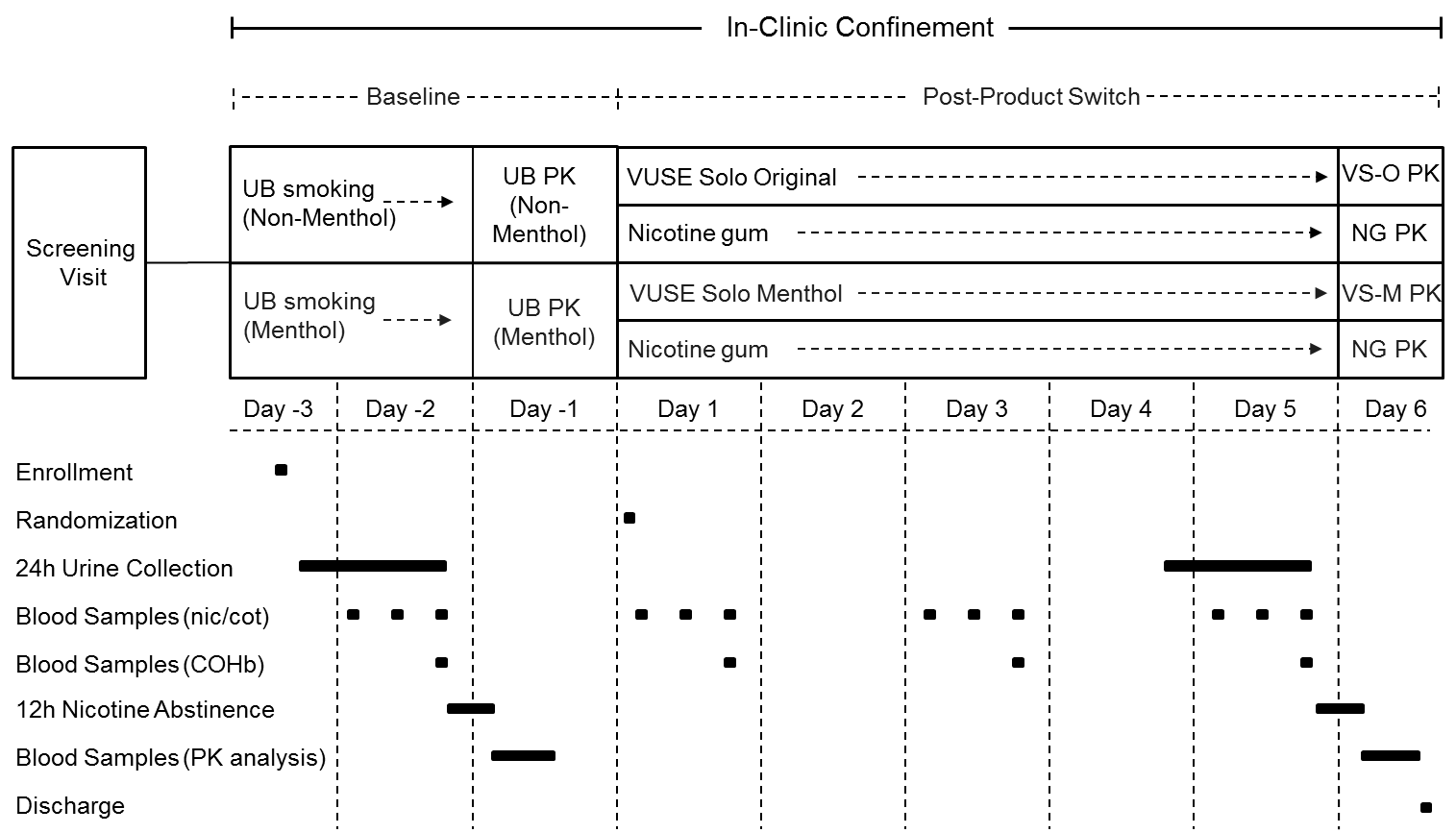


UB: Usual brand cigarette; PK: nicotine pharmacokinetics; VS-O: Vuse Solo Original; VS-M: Vuse Solo Menthol; NG: nicotine gum; nic/cot: nicotine/cotinine; COHb: carboxyhemoglobin
